# Exploring translational relevance of baseline and longitudinal metabolic profiling in the blood of ovarian cancer patients

**DOI:** 10.1038/s41698-025-01193-0

**Published:** 2026-01-24

**Authors:** Alexander Max Funk, Lisa Freitag, Franziska Maria Schwarz, Theresa Link, Sophie Jonas, Pauline Wimberger, Mareike Brieske, Anna Klimova, Triantafyllos Chavakis, Peter Mirtschink, Jan Dominik Kuhlmann

**Affiliations:** 1 Institute of Clinical Chemistry and Laboratory Medicine, University Hospital Carl Gustav Carus and Faculty of Medicine, Technische Universität Dresden, Fetscherstrasse 74, Dresden, Germany; 2 National Center for Tumor Diseases (NCT), NCT/UCC Dresden, a partnership between DKFZ, Faculty of Medicine and University Hospital Carl Gustav Carus, TUD Dresden University of Technology, and Helmholtz-Zentrum Dresden-Rossendorf (HZDR), Dresden, Germany; 3 Department of Gynecology and Obstetrics, Medical Faculty and University Hospital Carl Gustav Carus, Technische Universität Dresden, Dresden, Germany; 4 German Cancer Consortium (DKTK), Dresden and German Cancer Research Center (DKFZ), Heidelberg, Germany

**Keywords:** Ovarian cancer, Biomarkers, Tumour biomarkers, Metabolomics, Cancer metabolism

## Abstract

We conducted blood-based metabolomic profiling in ovarian cancer and determined its clinical relevance. NMR spectroscopy was performed on a total of *n* = 760 longitudinal plasma samples from *n* = 292 ovarian cancer patients, probing for *n* = 39 metabolites. At primary diagnosis, we revealed two distinguishable signatures, representing blood-based surrogates for a continuum of two metabolic states in ovarian cancer. These signatures shaped two subgroups of patients with differential surgical outcome and relapse risk (HR = 1.605, 95%CI:1.11-2.32, *p* = 0.009). Deconvolution of the metabolomic signatures identified acetoacetate, 3-hydroxybutyrate and alanine among the most relevant signature-determining metabolites. The acetoacetate^low^/3-hydroxybutyrate^low^/alanine^high^-profile was a strong predictor for superior clinical outcome, independently of FIGO stage and surgical outcome (HR = 0.471, 95%CI:0.236-0.942, *p* = 0.033). A strong relative decline of the ketone bodies in the course of therapy indicated adverse clinical outcome (acetoacetate: OS: HR = 2.22, 95%CI:1.08-4.55, *p* = 0.02). We propose a 3-metabolite blood-based signature in ovarian cancer that could be used for independent prediction of relapse risk and survival.

## Introduction

Ovarian cancer is the leading cause of death among women with gynecological malignancies. More than 70% of patients are diagnosed with advanced disease^1^. Standard treatment of advanced ovarian cancer consists of surgical debulking, aiming at macroscopic complete tumor resection and platinum/paclitaxel-based chemotherapy, followed by maintenance treatment with antiangiogenic bevacizumab^2–4^. In patients with homologous repair -deficient (HRD) tumors, defined by either a pathogenic breast cancer 1/2, early onset (*BRCA1/2*) mutation and/or genomic instability, a combination of bevacizumab with the poly ADP ribose polymerase inhibitor (PARPi) olaparib has been approved as maintenance therapy after response to first-line platinum-based chemotherapy^5^. Moreover, the PARPi niraparib is currently used as maintenance therapy without bevacizumab following response to first-line platinum-based treatment, regardless of HRD or *BRCA1/2* mutational status^6^. Despite these advances in standard treatment, the majority of patients with advanced ovarian cancer still face a poor overall prognosis, highlighting the urgent need for blood-based predictive biomarkers that would enable targeted therapies within the framework of precision medicine.

Metabolic reprogramming is a hallmark of cancer, including ovarian cancer, and plays a pivotal role in tumor initiation, progression, and metastasis^7,8^. A metabolomic research approach describes the quantitative measurement of multiple metabolites in cancer tissues or biological fluids, such as serum, plasma or urine^9^. The translational value of quantitative metabolomics as a tool for biomarker discovery and precision medicine has already been highlighted^9–11^. Changes in the metabolome, as an integrative downstream result of aberrant gene expression, are considered to more precisely capture biochemical activity and metabolic alterations, providing a functional snapshot of cancer cell metabolism compared to the transcriptome, or even the proteome^12^. Therefore, the combined application of quantitative metabolomics and bioinformatic modeling enables the resolution of distinct metabolic states of a patient’s tumor over the course of disease and may enable the stratification of patient subgroups with differential outcomes^11,13^.

Metabolomic reprogramming in ovarian cancer patients has already been studied, based on tumor biopsies or blood samples^9^. However, most of these investigations have been conducted in the context of early cancer detection^9^. In a case control study, for instance, it was proposed that the level of circulating lipid metabolites could complement the standard biomarker CA125 and may aid in discriminating between benign and malignant serous ovarian cancer^14^. Evidence is accumulating that blood-based metabolomic profiling has prognostic implications and could be useful for predicting recurrence risk in ovarian cancer^15–17^. However, the longitudinal progression of blood metabolomic profiles in response to primary debulking surgery and adjuvant chemotherapy, and its clinical relevance for ovarian cancer patients remains to be determined.

Using a preselected panel of 39 metabolites, associated with energy metabolism, oxidative stress response, mitochondrial dysfunction, and inflammation, we performed metabolomic profiling both at diagnosis and in longitudinal plasma samples of ovarian cancer patients, assessing its predictive and prognostic potential.

## Results

### Blood-based metabolomic profiling in ovarian cancer patients

**Fig. 1:**
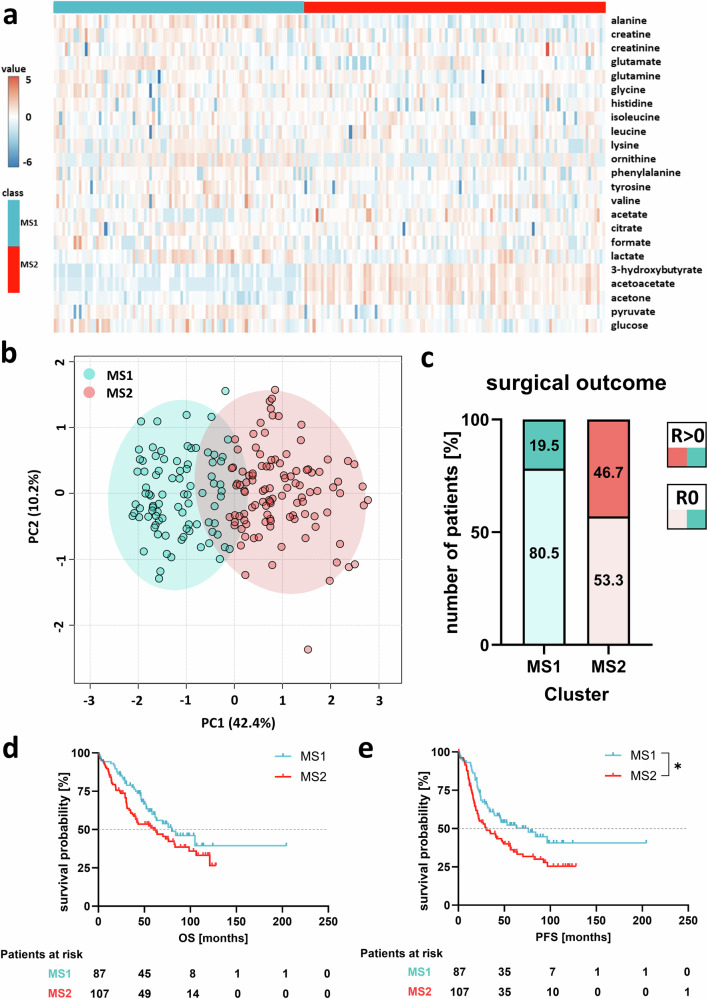
Blood-based metabolomic profiling in ovarian cancer patients. **a** Metabolite abundances across the identified metabolic signatures MS1 (cyan blue) vs. MS2 (red). **b** Principal component analysis, resulting in two clusters, representing MS1 (light red) and MS2 (cyan blue). **c** Distribution of surgical outcome status (macroscopic complete resection (R = 0) *vs*. any residual tumor (R > 0)) across the metabolic signatures MS1 and MS2. **d** Progression-free (PFS) and **e** overall survival (OS) of MS1 *vs*. MS2 patients. Statistical analysis was performed using the Log-rank (Mantel-Cox) test (* *p* < 0.05, ns *p* ≥ 0.05).

From the overall cohort (*n* = 292), NMR-based targeted metabolomic profiling was performed in all patients with available pre-treatment plasma at primary diagnosis (*n* = 194), using a panel of 39 metabolites involved in oxidative stress, mitochondrial dysfunction, and inflammation (Supplementary Table 1, Supplementary Fig. 1). Only metabolites with signals exceeding the limit of detection in ≥50% of samples (*n* = 23) were retained for downstream analyses. Using k-means clustering via MetaboAnalyst, metabolic signatures were identified, stratifying this analytical subcohort into metabolic signature 1 (MS1; *n* = 87) or metabolic signature 2 (MS2; *n* = 107; Fig. 1a, b). The observed clustering was independent of the plasma storage duration, patient age, or body mass index (BMI; Supplementary Fig. 2). MS1 was mainly characterized by lower levels of ketone bodies (acetoacetate, 3-hydroxybutyrate, acetone) and higher levels of specific amino acids (alanine, tyrosine, glutamate) and pyruvate, whereas MS2 was characterized by higher levels of ketone bodies and lower levels of these amino acids (Fig. 1a). Most of the patients’ clinicopathological parameters, including FIGO stage, grading, and *BRCA1/2* mutational status were balanced between MS1 vs. MS2 subgroups (Supplementary Fig. 2). However, patients assigned to MS2 at baseline showed higher CA125 levels (p = 0.0016) and a significantly higher incidence of incomplete tumor resection, suggesting that the metabolomic profile may serve as a surrogate marker of biological factors affecting resectability (p < 0.0001; Fig. 1c). Moreover, MS2 patients had a significantly shorter PFS (HR = 1.61, 95%CI:1.11-2.32, *p* = 0.009; median survival: 29.8 *vs*. 76.1 months) and a numerical trend for a shorter OS (HR = 1.431, 95%CI:0.964-2.123, *p* = 0.081, median survival: 60.2 *vs*. 79.6 months) compared to MS1 patients (Fig. 1d, e).

Conclusively, baseline signatures of mostly lipid and protein metabolites in the patient’s plasma shaped two subgroups of ovarian cancer patients (MS1 *vs*. MS2) with differential surgical outcome and survival.

### Identification of prognostically informative surrogates for the MS1/MS2 stratification at the resolution of individual metabolites

**Fig. 2:**
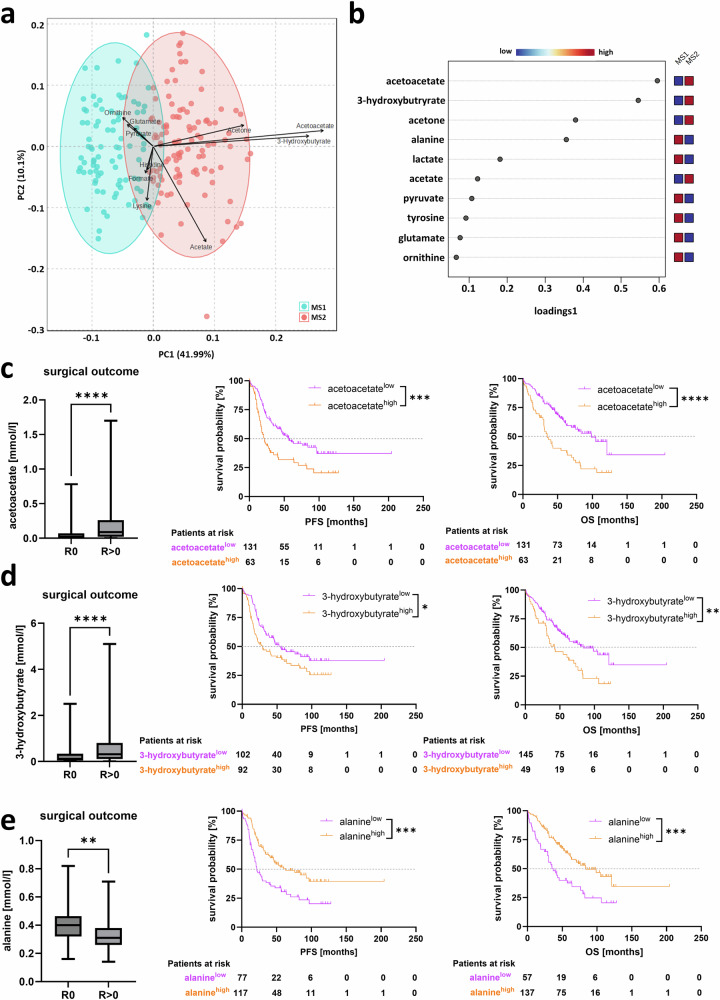
Deconvolution of metabolomic signatures M1 and M2. **a** Principal component analysis and **b** sparse PLS-DA algorithm, ranking individual metabolites with the greatest contribution to the observed clustering into MS1 *vs*. MS2. Progression-free (PFS), overall survival (OS) and surgical outcome status between high or low levels of (**c**) acetoacetate, cutoff:0.09 mmol/l (PFS/OS), **d** 3-hydroxybutyrate, cut-off:0.18 mmol/l (PFS), 0.46 mmol/l (OS) or (**e**) alanine, cutoff:0.35 mmol/l (PFS), 0.31 mmol/l (OS). Statistical analysis was performed using the Log-rank (Mantel-Cox) test (** *p* < 0.01, *** *p* < 0.001, **** *p* < 0.0001) and unpaired t-test (**** *p* < 0.0001).

Using sparse PLS-DA algorithm, we identified those metabolites of our panel that contributed most strongly to the observed clustering into MS1 *vs*. MS2 (Fig. 2a, b). Based on their individual loadings scores, the ketone bodies acetoacetate, 3-hydroxybutyrate and acetone, along with the amino acid alanine, emerged as the most relevant cluster-defining metabolites with the highest inter-patient variability (score >0.3; Fig. 2b) and exhibited significant differences in their median abundance between MS1 and MS2: acetoacetate, estimated difference (ED) = 0.09, *p* < 0.0001; 3-hydroxybutyrate, ED = 0.36, *p* < 0.0001; acetone, ED = 0.07, *p* < 0.0001; and alanine, ED = -0.13, *p* < 0.0001 (Supplementary Fig. 3).

We further investigated the clinical relevance of these metabolites and assessed their potential as blood-based biomarkers. The plasma concentrations of the individual ketone bodies (acetoacetate, 3-hydroxybutyrate, acetone) were unrelated to plasma storage duration, patient age, BMI or *BRCA1/2* mutational status (Supplementary Figs. 4–6). However, higher plasma concentrations of each ketone body predicted suboptimal surgical outcomes and were associated with advanced disease, as indicated by a higher FIGO stage and elevated serum CA125 concentrations (Fig. 2c, d Supplementary Figs. 4–6). Furthermore, higher concentrations of acetoacetate or 3-hydroxybutyrate at primary diagnosis predicted poor prognosis (acetoacetate, PFS: HR = 1.88, 95%CI: 1.23-2.87, *p* = 0.0008; median survival: 21.6 *vs*. 56.6 months; OS: HR = 2.23, 95%CI: 1.430-3.465, *p* < 0.0001; median survival: 35.2 *vs*. 98.7 months; 3-hydroxybutyrate, PFS: HR = 1.52, 95%CI:1.05-2.21, *p* = 0.024; median survival: 28.9 *vs*. 55.1 months; OS: HR = 1.80, 95%CI:1.13-2.89, *p* = 0.005; median survival: 42.0 *vs*. 83.9 months, Fig. 2c, d). A similar prognostic relevance of acetoacetate and 3-hydroxybutyrate was found by univariate regression analysis (Supplementary Table 2). The measured acetone concentrations were prognostically non-informative (Supplementary Fig. 6).

In contrast to ketone bodies, a lower baseline alanine concentration was associated with an increased likelihood of incomplete tumor resection, indicated advanced disease (FIGO III/IV, higher CA125 concentrations) and were associated with poor prognosis (PFS: HR = 0.512, 95%CI:0.35-0.76, *p* = 0.0003; median survival: 21.6 *vs*. 62.9 months; OS: HR = 0.478, 95%CI:0.30-0.76, *p* = 0.0002; median survival: 37.0 *vs*. 83.9 months; Fig. 2e; Supplementary Fig. 7; Supplementary Table 3).

**Fig. 3:**
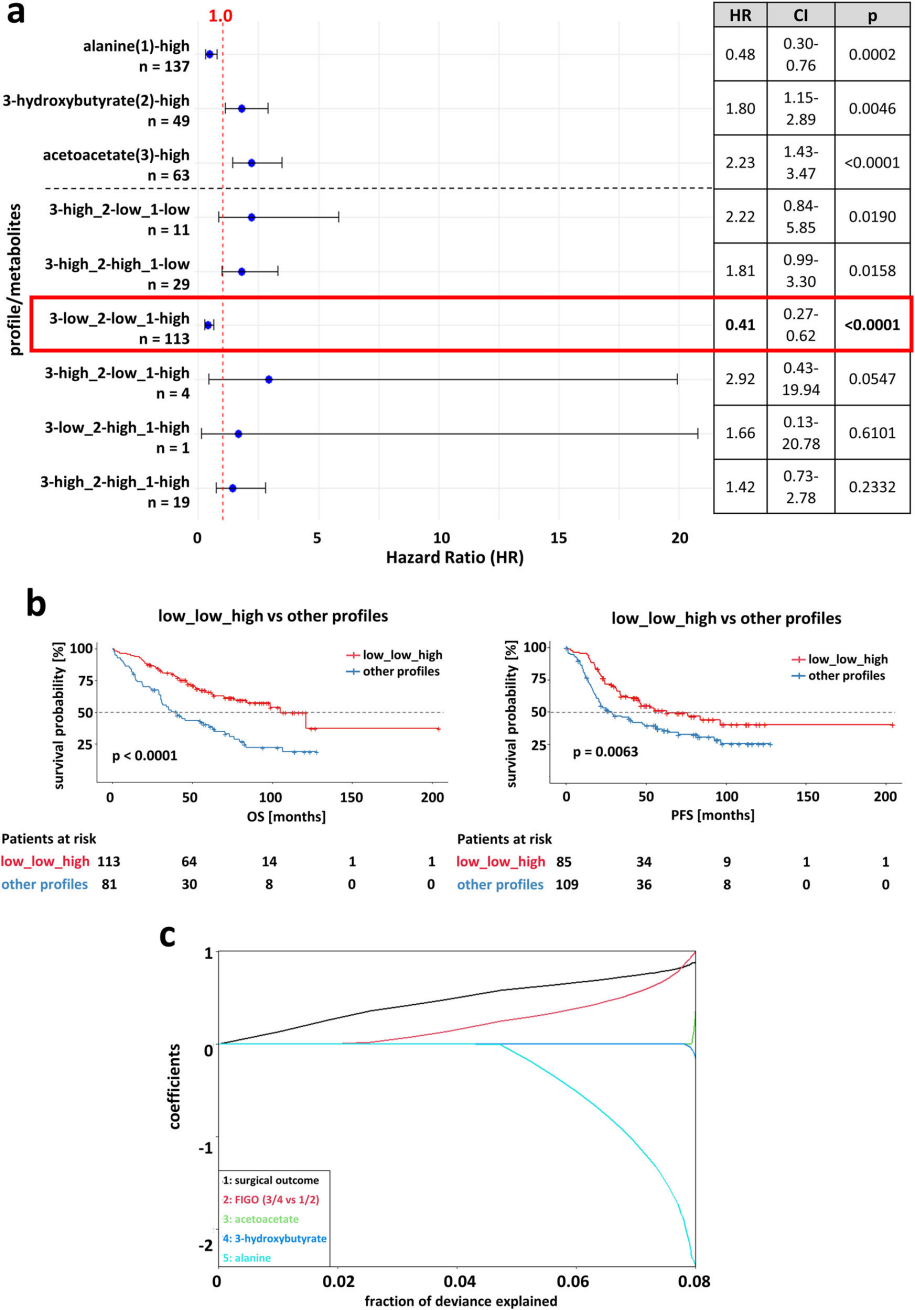
Identification of an optimized 3-metabolite signature. **a** Comparison of combined sets of metabolites (acetoacetate, 3-hydroxybutyrate, alanine) and the individual metabolites to the other possible profiles in order to identify the profile with the best clinical outcome (red box). **b** Progression-free (PFS) overall survival (OS) of patients with the “best profile” compared to other profiles. **c** Coefficient paths from a LASSO-penalized Cox regression model including the three selected metabolites and clinical covariates (FIGO stage, surgical outcome). Each curve represents the coefficient trajectory of one variable as a function of the fraction of deviance explained.

In order to determine an optimized prognostic profile, derived from the MS1/MS2 signatures, we performed combined analysis with all three metabolites, using dichotomization into “low” and “high” levels, according to the previously defined cutoff values (Fig. 2c-e). Cox proportional hazards models revealed that patients with acetoacetate^low^/3-hydroxybutyrate^low^/alanine^high^-profile were associated with the most pronounced estimated difference in clinical outcome (OS: HR = 0.413, 95%CI:0.27-0.62, *p* < 0.0001; median survival: 104.9 *vs*. 40.3 months), compared to the three metabolites alone or in any other combination (Fig. 3a). Log-Rank test led to a similar conclusion that patients with this “best-profile” had significantly longer PFS and OS (Fig. 3b), compared to those that didn’t match this signature. Ultimately, we assessed, whether our optimized 3-metabolite profile provides additional clinical value beyond already known clinical risk factors of ovarian cancer i.e. surgical outcome status and FIGO stage^18^. Overall, surgical outcome, FIGO stage, and alanine levels showed the strongest association with survival differences between patients, as determined by a LASSO-penalized Cox regression (Fig. 3c). According to multivariable analysis, adjusted for FIGO stage and surgical outcome status, the acetoacetate^low^/3-hydroxybutyrate^low^/alanine^high^-profile was an independent predictor for improved survival (HR = 0.471, 95%CI:0.236-0.942, *p* = 0.033; Table 1).

**Table 1:** Independent prognostic relevance of the identified 3-metabolite signature (acetoacetate, 3-hydroxybutarate, alanine)

*Variable*	*Outcome*	*β*	*exp(β)/HR*	*Lower bound 95%CI*	*Upper bound 95%CI*	*z*	*p-value*
***Surgical outcome***	***OS***	0.855	2.351	1.481	3.731	3.628	**0.0003**
***FIGO***		1.085	2.961	1.531	5.725	3.227	**0.0013**
***best profile***		-0.752	0.4712	0.236	0.942	-2.129	**0.0332**

Bold values mark *p* values < 0.05, highlighting statistically significant results.

Taken together, deconvolution of the metabolomic signatures MS1/MS2 at single metabolite resolution revealed the acetoacetate^low^/3-hydroxybutyrate^low^/alanine^high^-profile as an independent predictor of relapse risk and survival.

### Longitudinal progression of blood-based metabolic signatures during therapy and its clinical relevance

**Fig. 4:**
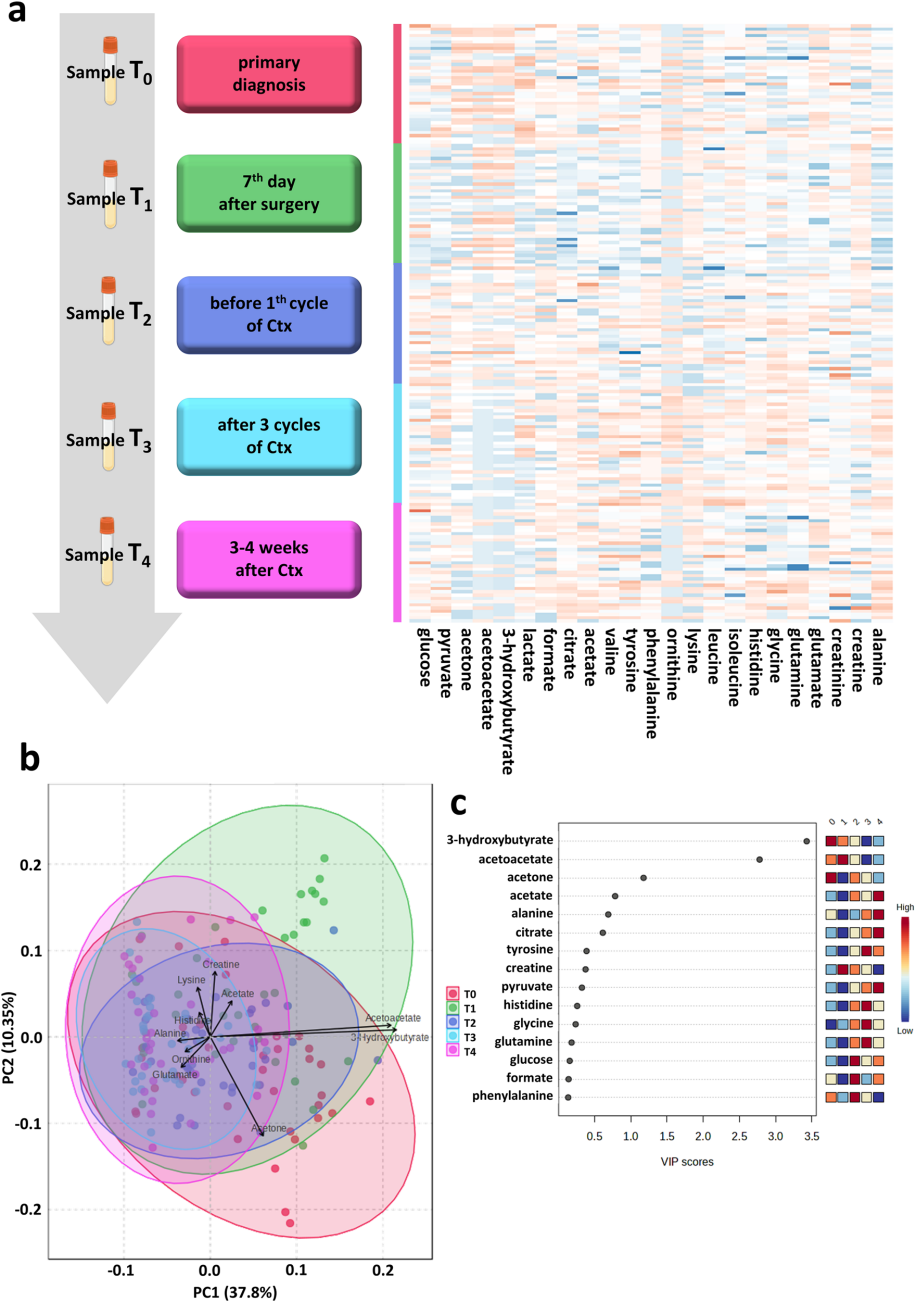
Longitudinal progression of blood-based metabolic signatures in the course of primary ovarian cancer treatment. **a** Longitudinal blood sampling strategy, resulting in five longitudinal plasma samples obtained in the course of primary treatment and framed by primary diagnosis and the completion of adjuvant platinum-based chemotherapy. Abundances of individual metabolites across the different time points of blood sampling are indicated by the heatmap. The illustration was created using images from Biorender.com. **b** Principal component analysis, showing temporal progression of the blood metabolomic profile. Vectors, representing to the most relevant cluster-determining metabolites are indicated. **c** Sparse PLS-DA algorithm, ranking individual metabolites with the greatest contribution to the temporal progression of the indicated clusters.

In order to study the temporal progression of metabolic signatures in the course of primary treatment, we profiled matched longitudinal serum samples, available from 37 of 292 study patients (Supplementary Fig. 1). In particular, the profile at primary diagnosis (T_0_) was compared with those obtained seven days after primary debulking surgery (T_1_), before the start of adjuvant chemotherapy (T_2_), after the first three cycles of chemotherapy (T_3_) and four weeks after the completion of chemotherapy (T_4_; Fig. 4a, b). PCA identified five distinct clusters that partially co-segregate with the respective blood sampling timepoints (Fig. 4b). Despite partial overlap between clusters, the PCA suggested a gradual, near -stepwise shift in metabolic profiles from primary diagnosis (T_0_) through to the end of chemotherapy (T_4_), indicating that both surgery and chemotherapy were associated with progressive changes in systemic metabolite composition. In addition, cluster dispersion was markedly broader at T_0_ and T_1_ compared to later timepoints, reflecting greater inter-individual variability in metabolite concentrations at diagnosis and after surgery, which became more homogeneous during chemotherapy (Fig. 4b). This suggests that several metabolites were altered in their concentrations at primary diagnosis and that the most noticeable change in the blood-based profiles occurred after primary debulking surgery, resulting in macroscopic complete tumor resection in most patients (*n* = 185/292; 63.3%).

**Fig. 5:**
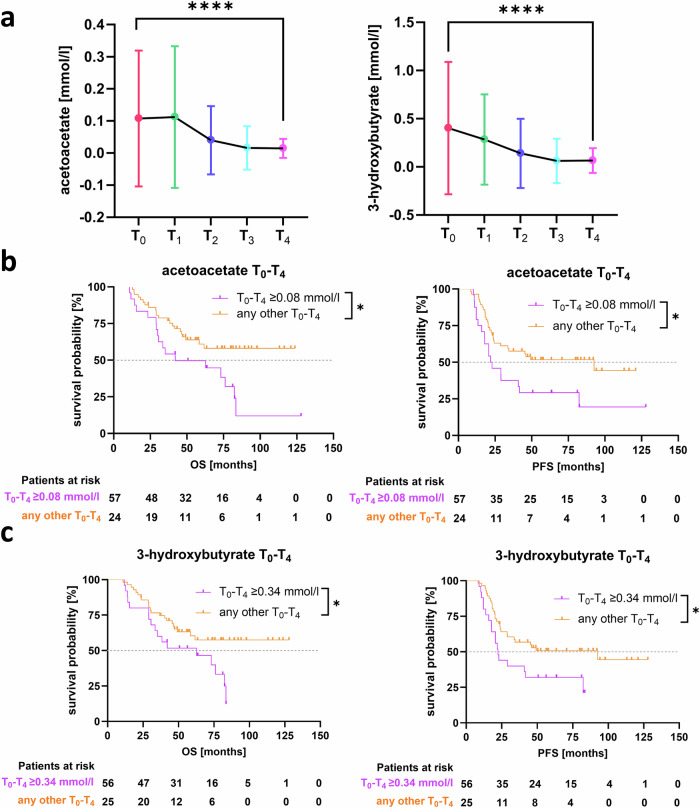
Prognostic relevance of longitudinal acetoacetate and 3-hydroxybutyrate levels. **a** Progression of acetoacetate and 3-hydroxybutyrate concentrations in the course of primary ovarian cancer treatment. Statistical analysis was performed using ANOVA with Tukey’s post hoc test (**** p_adj_<0.0001). Progression-free (PFS) and overall survival (OS) of patients with a high decrease in **b** acetoacetate and **c** 3-hydroxybutyrate levels between primary diagnosis (T_0_) and the completion of adjuvant chemotherapy (T_4_) compared to patients with any increase or a decrease of the respective ketone body (respective cutoffs are indicated in the figure legends). Statistical analysis was performed using the Log-rank (Mantel-Cox) test (* *p* < 0.05).

The overall shift of the five clusters was primarily driven by the vectors representing 3-hydroxybutyrate and acetoacetate, which pointed in the direction of the first principal component (PC), indicating that these two metabolites were associated with the most consistent changes in concentrations during primary treatment (Fig. 4c). Accordingly, both ketone bodies were significantly elevated at T_0_ and gradually decreased over the course of primary therapy (p < 0.0001, *p* < 0.0001, respectively; Fig. 5a). We asked next, whether the magnitude of this decrease in plasma ketone bodies under chemotherapy may represent a prognostic signal. To this end, we calculated the individual differences in acetoacetate and 3-hydroxybutyrate levels between primary diagnosis and the endpoint after completion of chemotherapy (T_0_-T_4_). This resulted in average T_0_-T_4_ differences of 0.08 mmol/l for acetoacetate (range: -0.15 mmol/l to 0.82 mmol/l) and 0.34 mmol/l for 3-hydroxybutyrate (range: -0.4 mmol/l to 5.01 mmol/l). Interestingly, patients showing a pronounced decline in acetoacetate or 3-hydroxybutyrate ( ≥ 0.08 mmol/l acetoacetate and ≥0.34 mmol/l 3-hydroxybutyrate) had a significantly shorter PFS (HR = 2.08, 95%CI:1.05-4.11, *p* = 0.013; median survival: 22.4 *vs*. 92.6 months; HR = 1.96, 95%CI:1.00-3.84, *p* = 0.023; median survival: 22.4 *vs*. 92.6 months) and OS (HR = 2.22, 95%CI:1.08-4.55, *p* = 0.012; HR = 2.07, 95%CI:1.02-4.20, *p* = 0.022; Fig. 5b, c). A similar result was reported from univariate Cox regression analysis (Supplementary Table 4). However, the concentration of ketone bodies at T_4_ alone was not prognostically informative (Supplementary Fig. 8). Thus, the quantitative nature of our NMR-based metabolite profiling permitted the definition of clear cutoff values for treatment-induced changes that distinguish patients at higher risk for early relapse and poorer survival.

Finally, we sought to identify additional dimensions in the temporal progression of metabolomic signatures. The T_1_ cluster, representing the profile obtained only seven days after debulking surgery, yet before the onset of chemotherapy (Fig. 4b), was characterized by a shift that appeared partially orthogonal to an imaginary central line connecting clusters T_0_ and T_4._ This shift was defined by several smaller vectors (e.g., acetate, creatine, ornithine, glutamate, lysine), representing combined directional components of PC1 and PC2 and likely reflects transient changes in the blood metabolic profile in response to debulking surgery. In fact, glutamate showed a transient decrease following surgery (Supplementary Fig. 9).

Taken together, primary ovarian cancer treatment was associated with near-gradual changes in the blood-based metabolomic profile over time, largely driven by a stepwise normalization of elevated acetoacetate and 3-hydroxybutyrate baseline concentrations. A pronounced relative decline in either of these two ketone bodies was associated with an adverse clinical outcome.

## Discussion

Ovarian cancer cells undergo metabolic reprogramming to maintain their tumorigenic phenotype^8^. In the present study, we explored the clinical relevance of baseline and longitudinal metabolomic profiling in plasma samples from ovarian cancer patients. Since our classification was based on a limited number of pre-selected metabolites, only a small fraction of the entire metabolomic complexity, present in patient plasma, could be captured. Nevertheless, we identified metabolomic signatures at primary diagnosis of ovarian cancer that stratified patients into two subgroups with differential surgical outcome and survival. This clustering into MS1 and MS2 was primarily driven by an inverse correlation between key metabolites of lipid and amino acid metabolism. After excluding critical confounders, such as patient age, BMI and sample storage time, we propose that MS1 and MS2 represent a blood-based surrogate for a continuum of two metabolic states in therapy naïve ovarian cancer, independent of the FIGO stage. While matched tumor tissue was not available for metabolomic profiling in this study, the robust associations between plasma metabolites and clinical outcomes support the presence of a tumor-associated metabolic state detectable in circulation. Given the known spatial heterogeneity of ovarian cancer metabolism, plasma profiling may offer a more integrative and clinically accessible readout than tissue-based analysis alone. Tumors of patients clustering into MS1, might be characterized by high glucose dependency and low metabolic flexibility, a phenotype that is often accompanied by enhanced alanine production. This phenomenon is largely driven by a reliance on glycolysis, in which glucose is converted to pyruvate. In the context of impaired mitochondrial oxidative metabolism, common in metabolically inflexible tumors, pyruvate is less likely to enter the tricarboxylic acid (TCA) cycle and is instead diverted toward alternative metabolic pathways. One such route involves its transamination to alanine, catalyzed by alanine transaminase (ALT), using glutamate as a nitrogen donor. This reaction helps not only to manage excess carbon flux from glycolysis but also serves as a mechanism to dispose of nitrogen, supporting cellular nitrogen homeostasis in highly proliferative cancer cells. Additionally, alanine can be exported from the tumor cell, functioning as a metabolic release valve to maintain redox balance and support hepatic gluconeogenesis for fuel regeneration. This shift toward alanine production has been observed in various glucose-addicted tumors, especially under hypoxic or low-glutamine conditions. Therefore, increased alanine production is a hallmark of tumors that favor aerobic glycolysis and have reduced capacity to oxidize pyruvate, reflecting their dependence on glucose and limited metabolic plasticity. Low acetoacetate and 3-hydroxybutyrate concentrations reflect reduced reliance on fatty acid oxidation (FAO). Glucose-dependent tumors may be more amenable to therapeutic intervention due to their decreased metabolic flexibility, which renders them vulnerable to disruptions in glycolytic flux or glucose availability.

MS2 likely reflects a metabolic state, characterized by high fatty acid oxidation^19^ and low protein metabolism, which is prognostically inferior to MS1. This may be due to the high metabolic flexibility of MS2 tumors, making them more adaptable and therefore harder to target with conventional therapies that rely on disrupting specific metabolic dependencies. A presumed reflection of the tumor metabolic state in the blood is further supported by a previous study, reporting that the concentration of 3,4-dihydroxybutyric acid and of several amino acid and sugar derivatives showed significant correlations between serum and ovarian cancer tissue^17^.

Given the known heterogeneity of ovarian cancer, it is plausible that these metabolic states potentially overlap with or even result from previously described ovarian cancer subtypes based on their transcriptional-^20–22^ or ‘multi-omic’-^23^ based identity. Moreover, as transcriptional subtyping in ovarian cancer has recently been conducted using 2752 metabolism-associated genes, it is conceivable that our blood-based MS2 classification reflects or is closely related with the recently proposed and prognostically unfavorable ‘C2 transcriptional ovarian cancer subtype’. This subtype is enriched for activation of lipid metabolism-related pathways, including fatty acid biosynthesis and sphingolipid metabolism^24^. Moreover, our findings suggest a potentially inverse regulation of lipid and protein metabolism in ovarian cancer.

As enhanced lipid synthesis or uptake promotes cancer growth, altered lipid metabolism is considered one of the most prominent metabolic hallmarks of cancer^25^. The omentum as a fat-rich organ represents a preferential metastatic site for ovarian cancer cells, suggesting that lipid uptake from this environment supports tumor expansion. Indeed, adipocytes have been shown to promote ovarian cancer metastasis and provide energy for rapid tumor growth via direct lipids transfer^26^. This may explain our observation that MS2 patients characterized by presumably high fatty acid oxidation, exhibit a more aggressive disease phenotype with a lower likelihood of achieving macroscopic complete tumor resection and worse clinical outcomes. Our findings are consistent with previous studies, reporting that ovarian cancer is frequently associated with alterations in lipid metabolism and that blood-based profiling of lipid metabolites may serve as a prognostic biomarker^27,28^.

Since the reproduction and prospective validation of an entire blood-based metabolomic signatures is challenging, we identified the ketone bodies acetoacetate/3-hydroxybutyrate and the amino acid alanine as minimally required surrogates to cover and even surpass the predictive and prognostic impact of the MS1/MS2 stratification. Ultimately, we further transformed these results into an optimized 3-metabolite signature with independent prognostic value, that could be easily implemented into routine laboratory diagnostic use in ovarian cancer patients. The clinical relevance of this finding is further corroborated by previous investigations, reporting that ovarian cancer exhibits a distinct metabolic signature, characterized by the accumulation of ketone bodies and hydroxybutyric acid metabolites in serum and tumor tissue that could be informative as a prognostic biomarker^17^.

Given the data-specific nature of PCA-based clustering, baseline-derived metabolic signatures were not applied to the longitudinal sub-analysis. Instead, a separate exploratory clustering was performed, followed by a focused analysis of individual metabolites, which showed stronger prognostic associations and greater reproducibility for potential clinical application. Longitudinal metabolomic assessment in the course of primary ovarian cancer treatment revealed a stepwise shifting of the blood-based metabolomic profile. The hypothesis that tumor metabolic states are likely mirrored in the blood was further supported by our observation that the most pronounced change in the blood-based metabolomic profile was observed after primary debulking surgery, by which a macroscopic complete tumor resection was achieved in the majority of patients. From there on, the identified clusters condensed and the inter-individual variability between the profiles strongly decreased, suggesting that altered metabolomic profiles re-converge to their physiological levels, even before onset of adjuvant chemotherapy. We further observed that the longitudinal progression of the metabolomic profiles was once again predominantly driven by the ketone bodies acetoacetate and 3-hydroxybutyrate, which had already defined the prognostic MS1/MS2 signature at primary diagnosis. Surprisingly, while a high level of the ketone bodies at primary diagnosis indicated adverse clinical outcome, a strong relative decline of the ketone bodies under surgery and adjuvant chemotherapy likewise indicated adverse outcome. This could be explained by the fact that in the majority of patients, the mean levels of acetoacetate and 3-hydroxybutyrate gradually decreased during therapy and converged to normal reference values. Therefore, patients with higher ketone body levels at T_0_ were inevitably associated with a higher relative decline during therapy than patients with lower T_0_ ketone body concentrations. However, it is known that ketone bodies have complex and pleiotropic roles that extend beyond their role in energy production. They can act as key signaling metabolites among the regulation of gene expression, inflammation or oxidative stress^29^. It was also reported that ketone bodies may enhance the efficacy of anti-cancer agents, including chemotherapeutic drugs^30^. Moreover, a ketogenic diet was shown to enhance the anticancer effect of immunotherapy^31^. This suggests that high levels of ketone bodies, although associated with poor prognosis at primary diagnosis, may favor chemotherapy response in our patients. This could explain why strong depletion of ketone bodies during chemotherapy was indicative of poor prognosis.

We performed plasma metabolomic profiling both at primary diagnosis and during treatment. Prognostic discrimination was markedly stronger at diagnosis, yielding superior predictive power compared to the longitudinal setting. Clinically, this suggests that our three-metabolite signature is more suitable for risk stratification at diagnosis than for monitoring treatment response. We envision that this signature could be integrated into existing prognostic frameworks to guide intensified first-line strategies, such as extended treatment durations, closer surveillance, or early initiation of immune checkpoint blockade or other emerging immunotherapies. All analyses were performed using standardized ^1^H NMR spectroscopy under certified laboratory-developed test protocols, ensuring high reproducibility and scalability for clinical application. While our study provides robust evidence for a clinically relevant metabolomic biomarker in ovarian cancer, it remains an exploratory discovery effort requiring external validation prior to clinical implementation, in line with consensus recommendations from ovarian cancer early-detection biomarker consortia including ROCkeTS and PREDICT^32,33^.

## Methods

### Patient characteristics

Patients were recruited and samples were obtained and processed at the Department of Gynecology and Obstetrics at the Technische Universität Dresden, Germany. Overall, *n* = 292 consecutive patients with histologically confirmed primary epithelial ovarian cancer, peritoneal cancer or cancer of the fallopian tube (primary diagnosis from 2013 to 2022) were included. The clinicopathological characteristics of the overall study cohort are summarized in Table 2. From the overall study population (*n* = 292), analytical subgroups were defined based on sample availability and study design. Baseline metabolic profiling was performed in all patients with pre-treatment plasma samples (*n* = 194), and longitudinal profiling in a separate subset with complete serial sampling across five time points (*n* = 37), as detailed in the study flow diagram (Supplementary Fig. 1). Patients received cytoreductive surgery with the aim of macroscopic complete tumor resection and the recommendation of platinum-based adjuvant chemotherapy, in line with national guidelines. Participation in clinical trials did not exclude patients. Overall survival (OS) and progression-free survival (PFS) were calculated from the date of primary diagnosis. The primary clinical endpoints were progression-free survival (PFS) and overall survival (OS). PFS was defined as the time from date of primary diagnosis to the date of first documented disease progression or death from any cause, whichever occurred first. OS was defined as the time from diagnosis to death from any cause. Patients without an event were censored at the date of last clinical follow-up. Survival times were calculated in months. The study had been approved by the Local Research Ethics Committee at the Technische Universität Dresden, Germany (reference number: EK74032013) and was performed in accordance with good clinical practice guidelines, national laws, and the Declaration of Helsinki. All study participants gave written informed consent. Ovarian cancer was reported in agreement with the WHOclassification of tumors derived from female genital tract and staging was documented according to the FIGO classification^34^, revised in 2014^35^. FIGO stage was reported according to the revised version for all patients who underwent primary surgery from 2014 onwards. In the absence of contraindications, patients with a tumor stage of FIGO III-IV were additionally treated with the antiangiogenic antibody bevacizumab, parallel to chemotherapy and as maintenance therapy. Surgical outcome was reported as macroscopically complete tumor resection *vs*. any residual tumor.

**Table 2:** Patient characteristics at primary diagnosis

Clinical variable	Total cohort	MS1	MS2	p-value (chi-square test)
**Number of patients**	292	87	107	
**Age [years] (%)**				
median (range)	63 (23–85)	62 (23–84)	63 (29–84)	
<60	117 (40.1)	37 (42.5)	48 (44.9)	0.745
>=60	175 (59.9)	50 (57.5)	59 (55.1)	
**BMI (%)**				
<25	123 (43.8)	41 (48.8)	47 (44.3)	0.539
>=25	158 (56.2)	43 (51.2)	59 (55.7)	
**FIGO (%)**				
I/II	63 (21.6)	30 (34.5)	27 (25.2)	0.160
III/IV	229 (78.4)	57 (65.5)	80 (74.8)	
**Surgical debulking (%)**				
residual disease	107 (36.7)	17 (19.54)	50 (46.73)	**<0.0001**
macroscopic complete resection	185 (63.3)	70 (80.46)	57 (53.27)	
**Histology (%)**				
serous	233 (79.8)	70 (80.5)	77 (72.0)	0.170
non-serous	59 (20.2)	17 (19.5)	30 (28.0)	
**Tumortype (%)**				
HGSOC	217 (74.3)	62 (32.0)	64 (33.0)	0.251
LGSOC	16 (5.5)	4 (2.1)	7 (3.6)	
other tumortypes	59 (20.2)	21 (10.8)	36 (18.6)	
**BRCA1/2 mutational status (%)**				
*BRCA1/2* *wt*	97 (33.2)	31 (35.6)	24 (22.4)	0.965
*BRCA1/2mut*	47 (16.1)	19 (21.8)	15 (14.0)	
unknown	148 (50.7)	37 (42.5)	68 (63.6)	
**Progression-free survival**				
median (range)	28 (1–204 months)	42 (1–204 months)	26 (0–128 months)	
progression/death (%)	185 (63.3)	43 (49.4)	71 (66.4)	**0.017**
no progression/death (%)	107 (36.7)	44 (50.6)	36 (33.6)	
**Overall survival**				
median (range)	46 (1–204 months)	50 (1–204 months)	42 (0–128 months)	
dead (%)	152 (52.0)	38 (43.7)	61 (57.0)	0.065
alive (%)	140 (47.9)	49 (56.3)	46 (43.0)	
**CA125 [U/ml]**				
median (IQR)	381 (6.5–29800.0)	171 (6.5–8911)	480.9 (6.7–14207)	
<65 (%)	66 (23.7)	32 (39.0)	18 (17.8)	**0.002**
>=65 (%)	212 (76.3)	50 (61.0)	83 (82.2)	

Bold values mark *p* values < 0.05, highlighting statistically significant results.

*MS1* metabolic signature 1, *MS2* metabolic signature 2, p-values were calculated by chi-square test between MS1 and MS2.

### Blood sampling and plasma separation

Plasma preparation was performed as described previously by us^36^. Briefly, blood was drawn in EDTA S-Monovettes (9 mL; Sarstedt AG & Co., Nümbrecht, Germany), stored at 4 °C and processed within 4 h to avoid blood cell lysis. Each sample was centrifuged for 8 min at 3.300 × *g* and subsequently, 3–4 mL of the upper plasma phase were removed and frozen at -80 °C in aliquots of 2 mL until further processing. Unnecessary freeze-thaw cycles were avoided. For analysis, samples were thawed on ice and were immediately processed after complete thawing. The samples were blinded, so that neither time of blood drawing nor any other patient-related information could be disclosed during investigation.

### Targeted blood-based metabolomic profiling

^1^H NMR spectroscopy analysis was performed, according to established protocols^37^. The frozen plasma samples were thawed at room temperature for approximately 30 min. Afterwards, samples were mixed 1:1 with phosphate buffer, containing the internal reference trimethylsilylpropanoic acid (TMSP), resulting in a final volume of 600 µL. The mixture was then immediately transferred to an NMR sample tube. All NMR measurements were performed on a Bruker 600 MHz Avance III Neo, equipped with a BBI Probe and a Bruker SampleJet, which includes a cooling system for sample storage at 4 °C. The samples were measured at 310 K and a full quantitative calibration was completed before the measurement. All measurements followed the Bruker in-vitro diagnostics (IVDr) SOPs and methods. All data were processed automatically using Bruker TopSpin 4.1.1 and ICON NMR. Automatic metabolite and lipoprotein reports were obtained using Bruker IVDr B.I. Methods Plasma (B.I.Quant-PS, v2.0.0) and Bruker IVDr Lipoprotein Subclass Analysis (B.I.LISA, v1.0.0).

### Statistical analysis

Statistical analysis was performed with GraphPad Prism version 10.2.3 (GraphPad Software, La Jolla, CA, USA), and R version 4.4.1^38^ adapted from^39–41^. Graphical visualization was conducted using MetaboAnalyst 6.0^42^ and GraphPad Prism v10.2.3. P-values < 0.05 were considered statistically significant. All confidence intervals (CIs) were specified as 95% CI. Differences of the mean were calculated by ANOVA with Tukey’s post hoc test to account for multiple testing. MetaboAnalyst unsupervised principal component analysis (PCA) and K-means clustering was used for exploratory analysis of the metabolite profiles. To further identify the most relevant cluster-determining metabolites, we used sparse partial least squares discriminant analysis (sPLS-DA) and compared the absolute values of their respective loadings. For normalization, all data were mean-centered and a base-10 logarithmic transformation was applied within MetaboAnalyst. In some samples, metabolite concentrations remained below the limit of detection, a common occurrence in NMR-based metabolomic analyses. Consequently, metabolites with more than 50% of the measurements recorded as zero were excluded from further analysis. Optimal cutoff levels for each biomarker were determined using the surv_cutpoint() function from the survminer R package. Association between individual metabolites or metabolite signatures with clinicopathologic parameters were compared using chi-square test and unpaired t-test. Cox proportional hazards model with multiple variables was performed with progression-free survival (PFS) and overall survival (OS) as outcome variables and was adjusted for established clinical risk factors, i.e., age, CA125, FIGO stage, and surgical outcome. Survival analyses were performed using Kaplan-Meier curves. The significance of survival differences was assessed using the log-rank (Mantel-Cox) test and hazard ratios (HR) were calculated using Cox proportional hazards regression. PFS and OS were analyzed as continuous time-to-event variables in all statistical models. For the analysis of clinicopathological parameter distributions across metabolic signatures, age and body mass index (BMI) were treated as continuous variables, while FIGO stage, tumor type, CA-125 levels, and BRCA1/2 mutation status were assessed as categorical variables. In analyses evaluating associations between metabolite levels and clinical characteristics, all covariates were used in categorized form. LASSO penalized Cox regression^43^ implemented via the glmnet package in R^44^, was used to investigate interaction of the metabolites of interest with FIGO stage and surgical outcome and to account for overfitting.

## Supplementary information

Supplementary Material.

## Data Availability

All relevant data has been included into the manuscript. Raw data can be obtained upon reasonable request to the corresponding author.
