# Supplementary material for: Exploring translational relevance of baseline and longitudinal metabolic profiling in the blood of ovarian cancer patients
Source: NPJ Precis Oncol. 2026 Jan 24;10:45. doi: 10.1038/s41698-025-01193-0 (PMC12852794; doi:10.1038/s41698-025-01193-0)
Supplement: Supplementary file 1 — Supplementary Material. [file 41698_2025_1193_MOESM1_ESM.pdf]

Supplementary Material

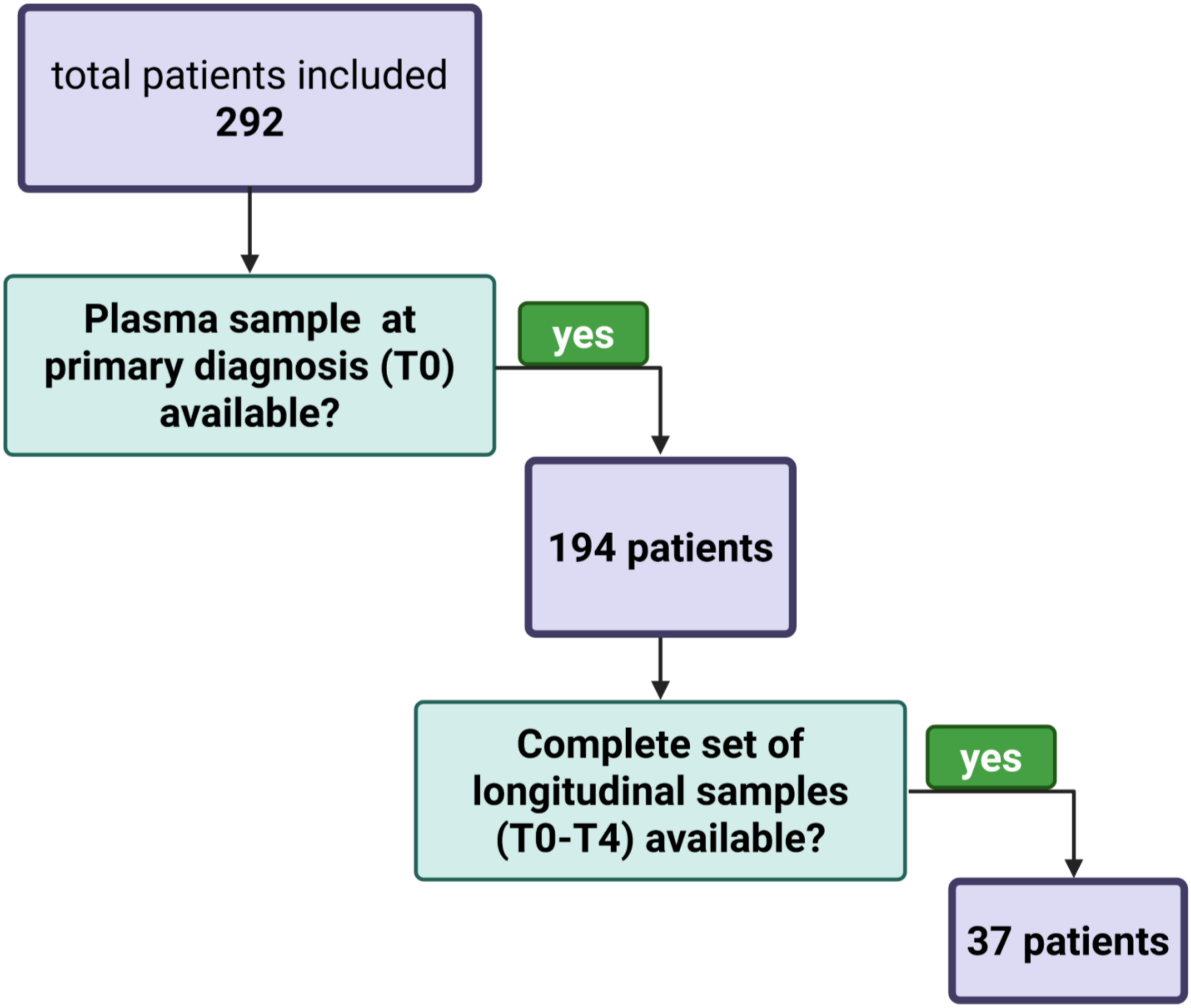

Supplementary Figure 1: Schematic representation of patients included in the study and subsequent selection. Samples are available from primary diagnosis (T_0), after surgery (T_1), before chemotherapy (T_2), within chemotherapy (T_3) and after completion of chemotherapy (T_4). The illustration was created using biorender.com.

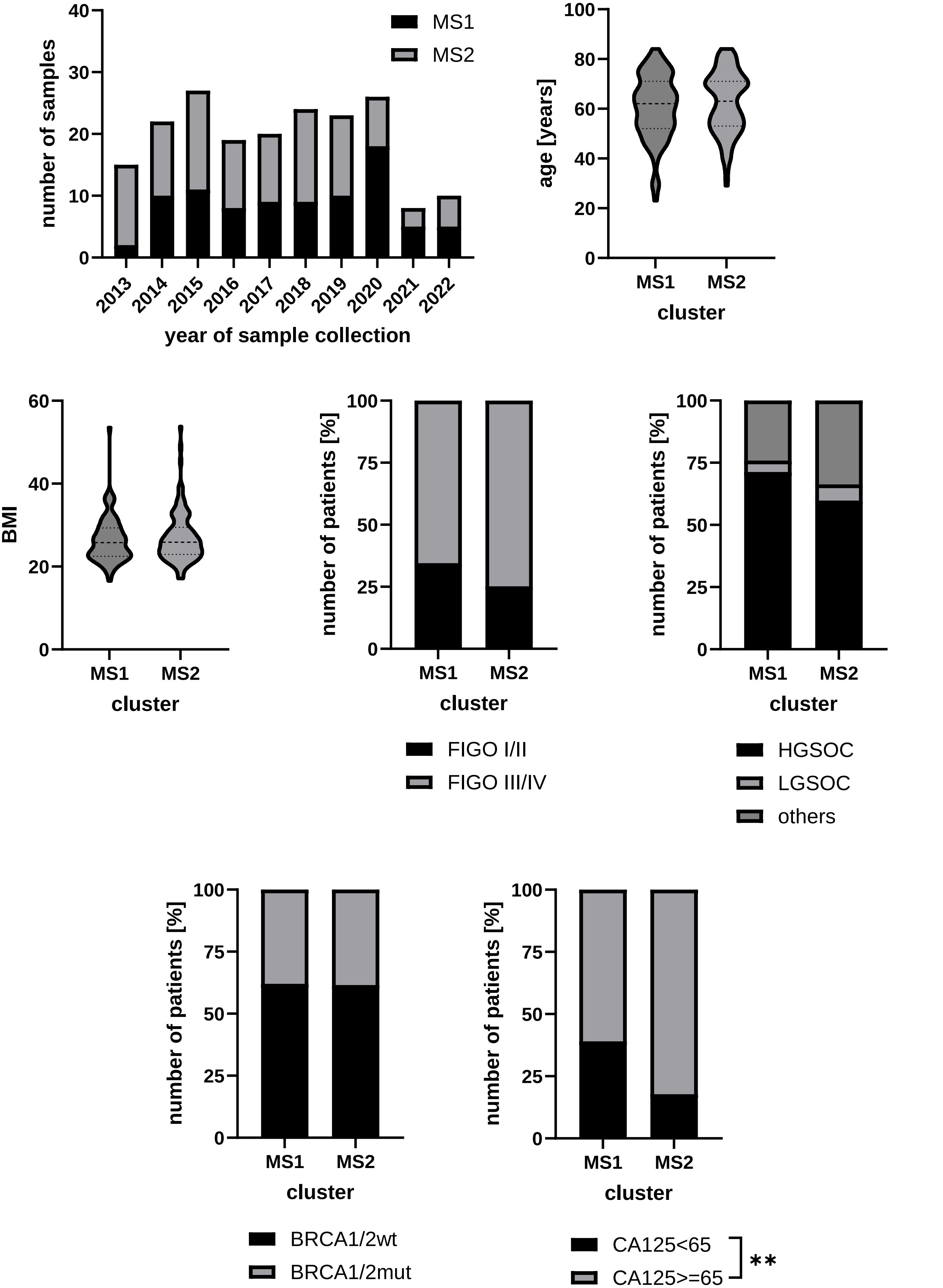

Supplementary Figure 2: Distribution of clinicopathological parameters among the identified blood-based metabolic signatures. MS1: metabolic signature 1, MS2: metabolic signature 2; all statistical comparisons were performed using an unpaired t-test or chi-square test, ** $p<0.01$.

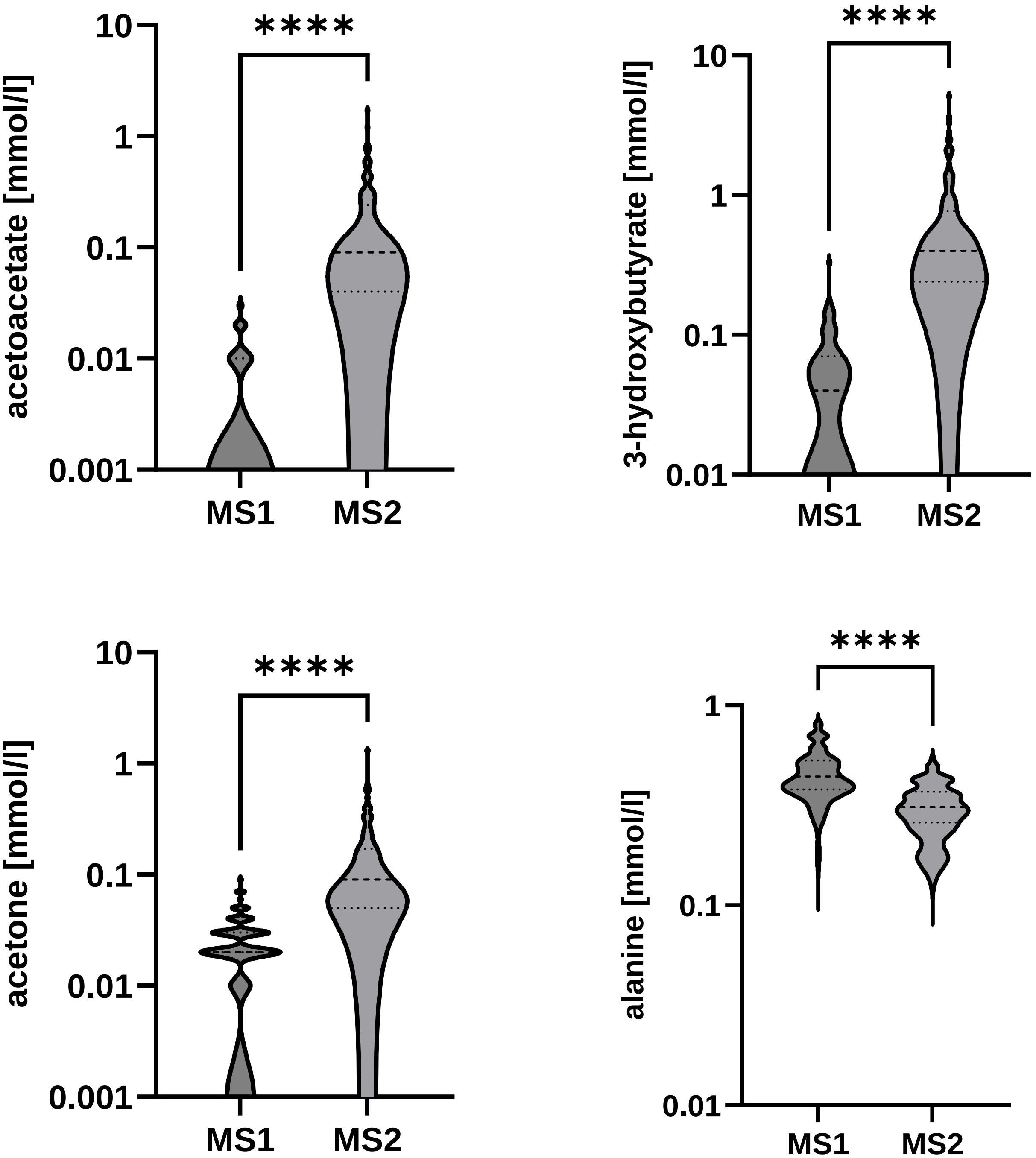

Supplementary Figure 3: Distribution of plasma acetocetate, 3-hydroxybutyrate, acetone and alanine levels among the identified blood-based metabolic signatures. MS1: metabolic signature 1, MS2: metabolic signature 2; statistical analysis was performed using an unpaired t-test; **** p<0.0001.

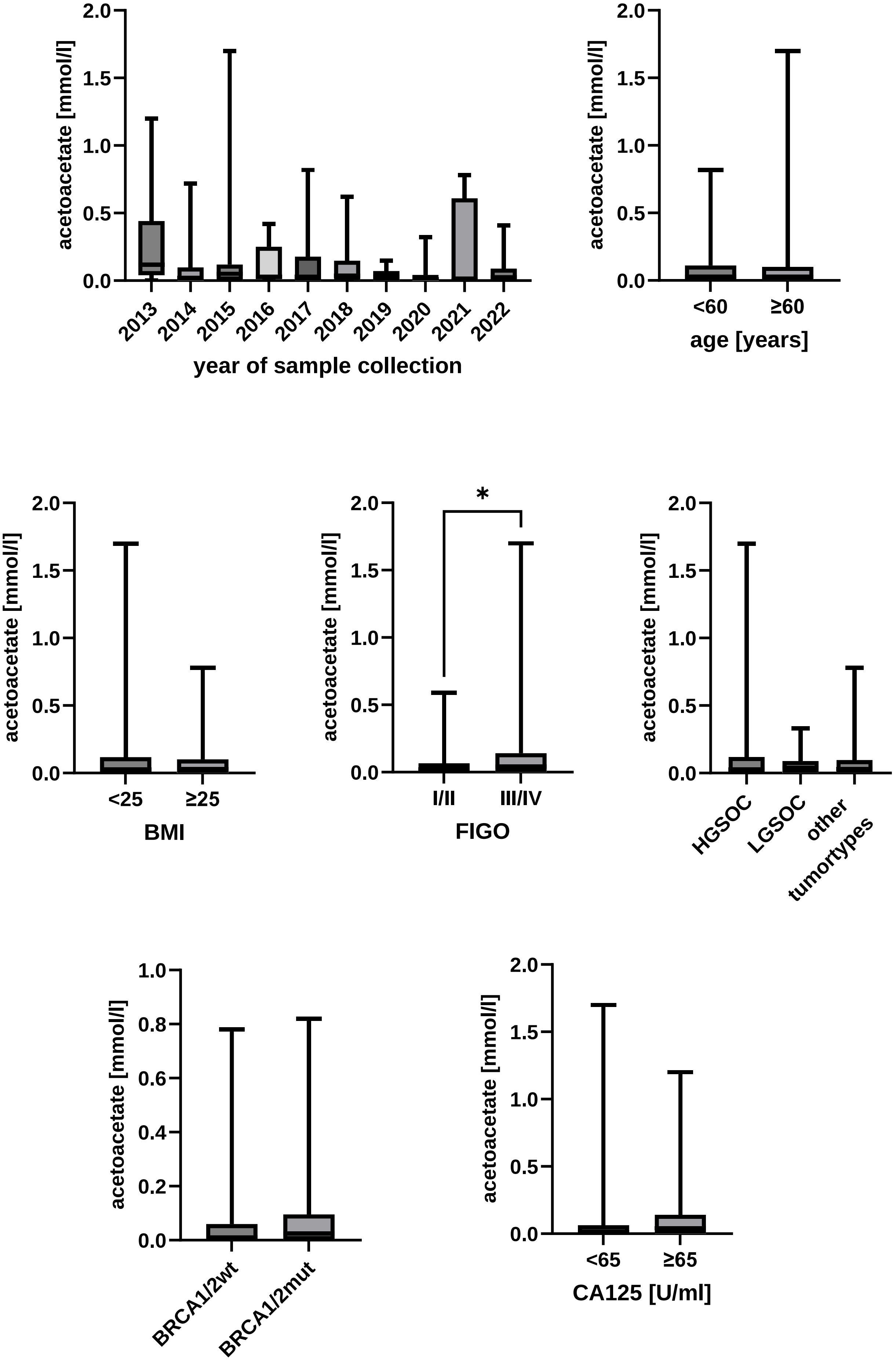

Supplementary Figure 4: Association of plasma acetoacetate levels with clinicopathological parameters. Statistical analysis was performed using one-way ANOVA with Tukey's post hoc test or an unpaired t-test; * $p<0.05$.

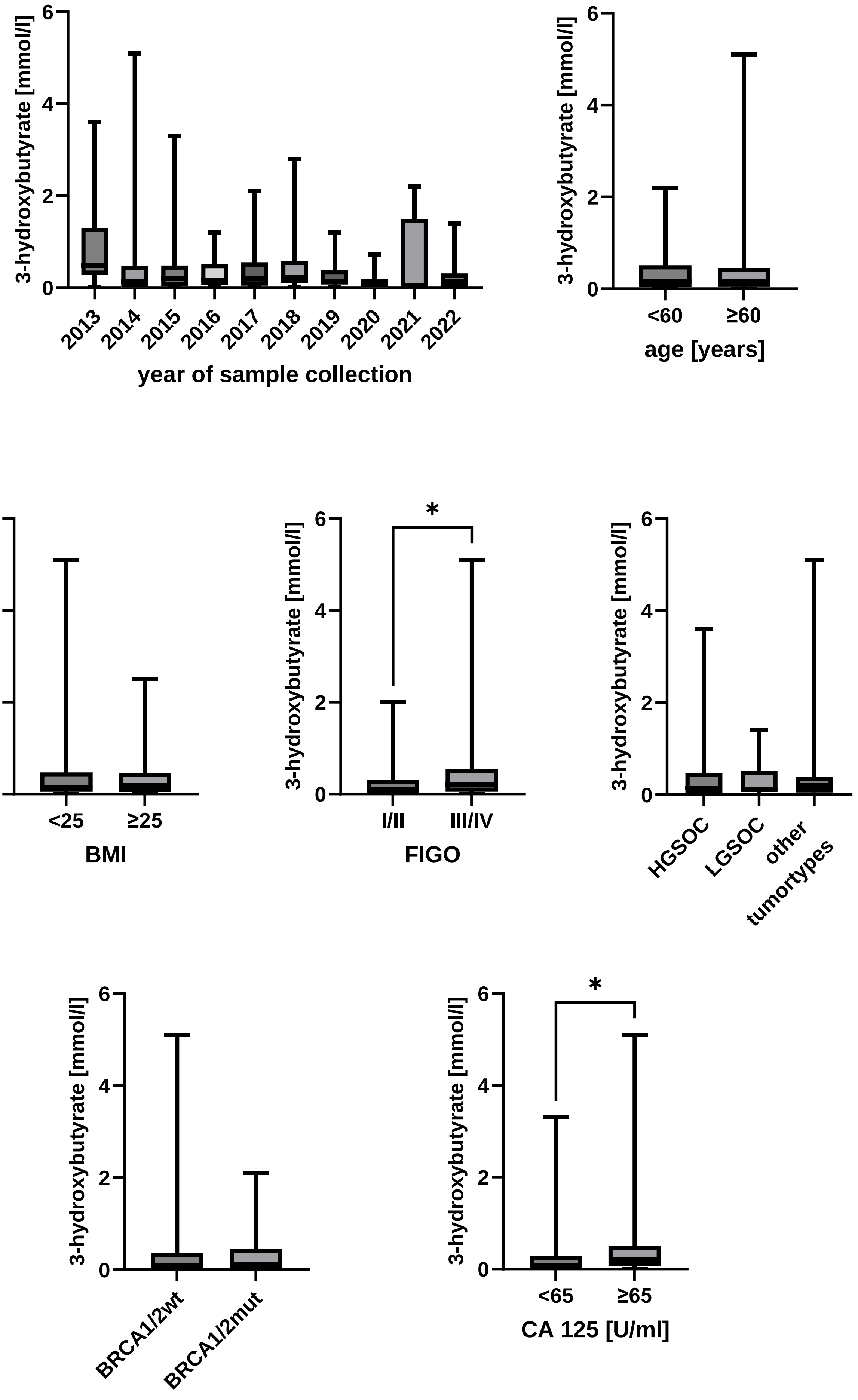

Supplementary Figure 5: Association of plasma 3-hydroxybutarate levels with clinicopathological parameters. Statistical analysis was performed using one-way ANOVA with Tukey's post-hoc test or an unpaired t-test; * $p<0.05$.

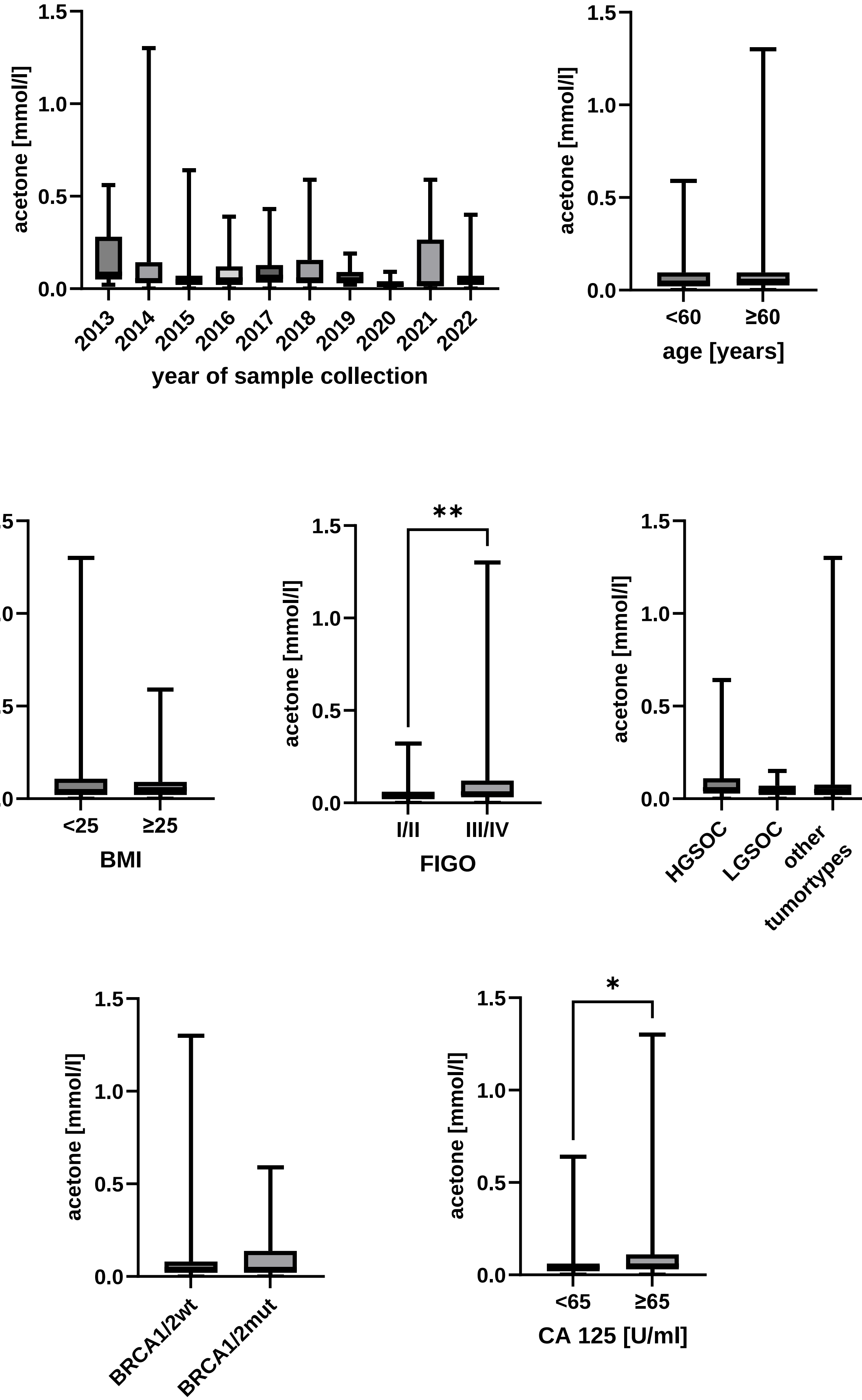

Supplementary Figure 6: Association of plasma acetone levels with clinicopathological parameters. Statistical analysis was performed using one-way ANOVA with Tukey's post-hoc test or an unpaired t-test; * p<0.05, ** p<0.01.

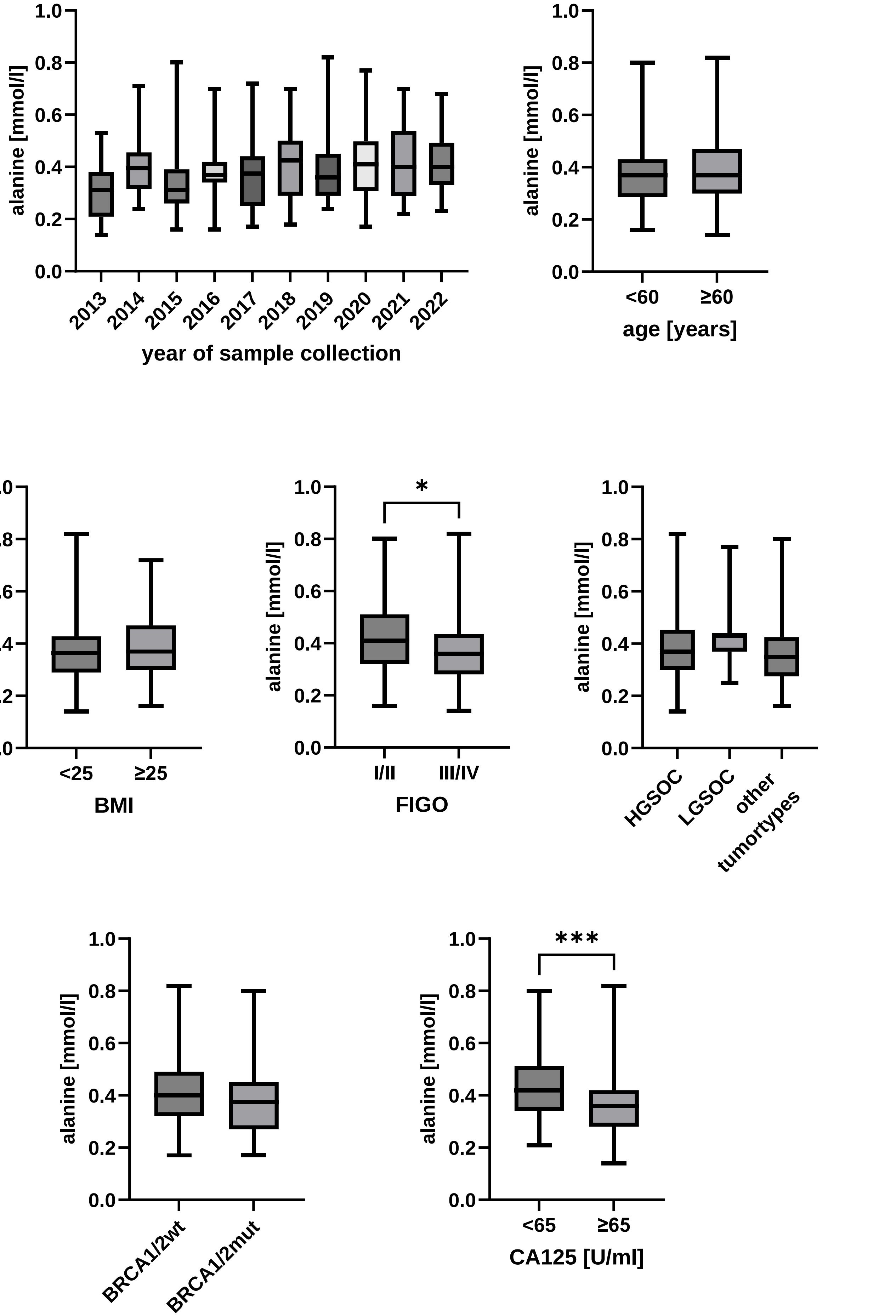

Supplementary Figure 7: Association of plasma alanine levels with clinicopathological parameters. Statistical analysis was performed using one-way ANOVA with Tukey's post-hoc test or an unpaired t-test; * $p<0.05$, *** $p<0.001$.

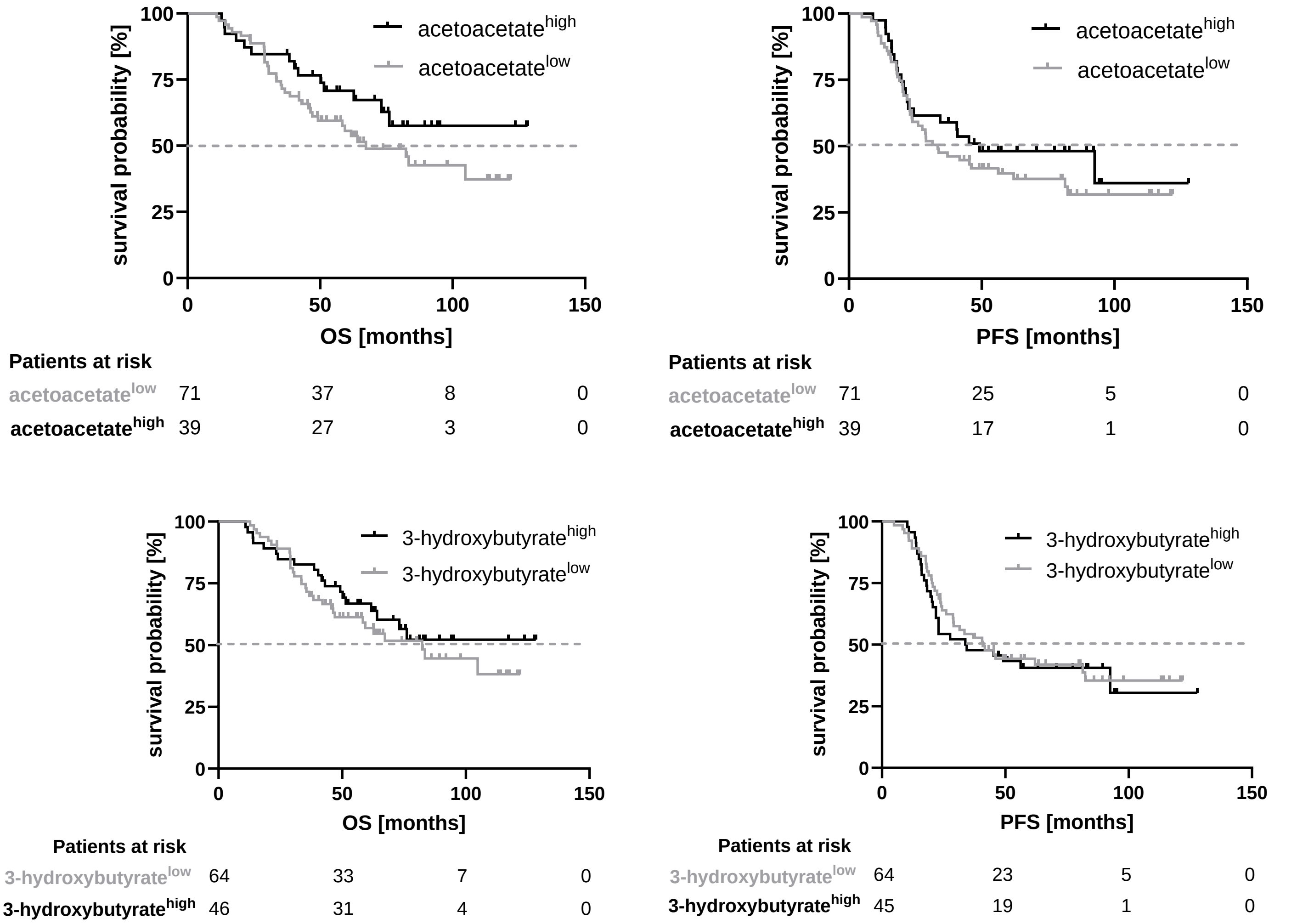

Supplementary Figure 8: Prognostic relevance of ketone body levels (acetoacetate, 3-hydroxybutyrate) after the completion of chemotherapy. Survival analyses were performed using Kaplan-Meier curves. All comparisons were non-significant as determined by the log-rank (Mantel-Cox) test.

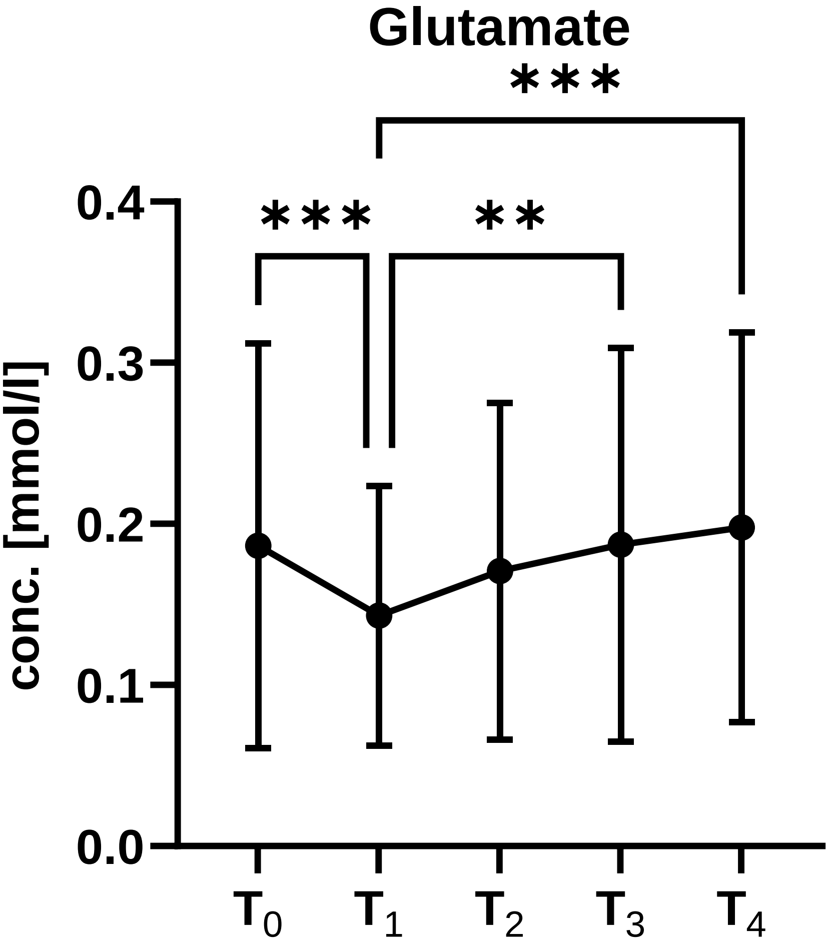

Supplementary Figure 9: Progression of glutamate levels in the course of primary ovarian cancer treatment. Statistical analysis was performed using ANOVA with Tukey's post-hoc test; ** padj<0.01, *** padj<0.001.

Supplementary Table 1: List of metabolites.

Category	Compound	LOD [mmol/l]
alcohols and derivatives	ethanol	0.10
amines and derivatives	trimethylamine-N-oxide	0.08
amino acids and derivatives	2-aminobutyrate	0.05
	alanine	0.02
	asparagine	0.05
	creatine	0.01
	creatinine	0.01
	glutamate	0.05
	glutamine	0.02
	glycine	0.01
	histidine	0.02
	isoleucine	0.03
	leucine	0.01
	lysine	0.04
	methionine	0.05
	N,N-dimethylglycine	0.01
	ornithine	0.02
	phenylalanine	0.03
	proline	0.05
	sarcosine	0.01
	threonine	0.04
	tyrosine	0.03
	valine	0.03
carboxylic acids	2-hydroxybutyrate	0.15
	acetate	0.01
	citrate	0.03
	formate	0.02
	lactate	0.03
	succinate	0.01
essential nutrient	choline	0.05
Keto acids and derivatives	2-oxoglutarate	0.02
	3-hydroxybutyrate	0.02
	acetoacetate	0.01
	acetone	0.01
	pyruvate	0.03
sugars and derivatives	D-galactose	0.11
	glucose	0.54
	glycerol	0.08
sulfones	dimethylsulfone	0.01
technical controls	Ca-EDTA	0.5
	K-EDTA	0.5

Supplementary Table 2: Prognostic relevance of acetoacetate and 3-hydroxybutyrate (univariate cox regression).

Metabolite	*Outcome*	*β*	*exp(β)/HR*	*Lower bound 95%CI*	*Upper bound 95%CI*	*z*	*p-value*
acetoacetate	***PFS***	0.6366	1.8901	1.293	2.764	3.285	**0.0010**
	OS	0.8060	2.2390	1.505	3.331	3.978	**6.96e-05**
3-hydroxybutyrate	***PFS***	0.4221	1.5252	1.054	2.208	2.238	**0.0252**
	OS	0.5916	1.8069	1.193	2.738	2.791	**0.0053**

Supplementary Table 3: Prognostic relevance of alanine (univariate cox regression).

Metabolite	*Outcome*	*β*	*exp(β)/HR*	*Lower bound 95%CI*	*Upper bound 95%CI*	*z*	*p-value*
alanine	***PFS***	-0.6727	0.5104	0.3526	0.7387	-3.565	**0.0004**
	OS	-0.743	0.4757	0.3174	0.7128	-3.601	**0.0003**

Supplementary Table 4: Prognostic relevance of longitudinal acetoacetate and 3-hydroxybutyrate levels (T_0-T_4).

Metabolite	*Outcome*	*β*	*exp(β)/HR*	*Lower bound 95%CI*	*Upper bound 95%CI*	*z*	*p-value*
acetoacetate	***PFS***	0.6823	1.9784	1.092	3.584	2.25	**0.0244**
	OS	0.7372	2.0902	1.107	3.945	2.275	**0.0229**
3-hydroxybutyrate	***PFS***	0.6521	1.9195	1.056	3.489	2.139	**0.0325**
	OS	0.6840	1.9819	1.05	3.739	2.112	**0.0347**
